# Functional Outcomes of Secondary Procedures in Upper Extremity Replantation and Revascularization

**DOI:** 10.7759/cureus.5164

**Published:** 2019-07-17

**Authors:** Nasir Khan, Mamoon Rashid, Haroon Ur Rashid, Saad Ur Rehman Sarwar, Usama Khalid Choudry, Mariam Khurshid

**Affiliations:** 1 Plastic Surgery, Shifa International Hospital, Islamabad, PAK; 2 General Surgery, Shifa International Hospital, Islamabad, PAK; 3 Dermatology, Pakistan Air Force Hospital, Islamabad, PAK

**Keywords:** replantations, revascularisation, secondary procedures, functional outcomes

## Abstract

Introduction

Traumatic amputation of the upper limb has significant associated morbidities and disabilities. After successful replantation surgery, the micro-surgeons’ tasks are far from over. The replanted and revascularized segments have numerous functional restrictions and need various corrective secondary procedures. The aim of our study was to compare the functional results after secondary procedures by administering the Quick Disability of the Arm, Shoulder, and Hand (QuickDASH) questionnaire to patients who had successful upper limb replantation and revascularization.

Materials and methods

This prospective observational study involved 40 patients who had a partial or complete amputation of the upper limb and underwent secondary procedures to correct function after successful replantation and revascularization surgery. The patients’ functional outcomes after various secondary procedures were recorded using the QuickDASH questionnaire.

Results

The mean QuickDASH score for thumb injuries was 42.3 pre-surgery but improved to 29.5 after secondary procedures, which was statistically significant (CI 11.12-14.87, p<0.01). The mean difference in the QuickDASH scores for finger injuries was also statistically significant: 45.5 preoperation and 33.7 postoperation (CI 9.89-13.70, p<0.01). For wrist injuries, the mean QuickDASH score was 52.8 presurgery and was 46.3 postoperatively (CI 1.81-6.58, p=0.0023). The QuickDASH scores of the patients with arm and forearm injuries showed no statistically significant improvement, with a preoperation score of 58.3 declining to 55.2 (p=0.98). The overall replantation and revascularization scores were 49.725 and 41.175 pre and postoperation, respectively (CI 8.35-8.75, p<0.01).

Conclusion

The study finds that the level and mechanism of injury are important predictors of the functional outcomes of the replantation and revascularization of amputated upper-limb appendages. Most replanted and revascularized upper limbs have numerous functional limitations, and achieving good functional results requires one or more secondary procedures, whose type depends on various factors such as the injury type and mechanism. The QuickDASH results for functional outcomes before and after secondary procedures indicate that it is an easy-to-use, reliable, and effective measure of functional outcomes.

## Introduction

Throughout history, humankind has dreamed of restoring youth and even life to the dead. Modern plastic surgery has been able to restore life to lifeless extremities through replantation [1]. Traumatic upper-limb amputations are more common than lower-limb amputations. Due to the occurrences of injury in remote areas and the lack of awareness, personal protective equipment, and microsurgery centers, most patients in underdeveloped countries do not receive replantation or revascularization, resulting in lifelong disabilities. Upper-limb prostheses are much more expensive and less efficient than lower-limb prostheses. The functional and psychological impacts of amputation of the upper extremities can severely alter the patients’ quality of life [2].

Replantation is the process of attaching a completely amputated body segment by anastomosing both the arterial inflow and the venous outflow. Revascularization is reattaching an incompletely amputated part with compromised blood supply regardless of tissue type [3]. The decision to perform revascularization or replantation for traumatic injuries depends on several factors, including ischemia time, mechanism of injury, level of amputation, patients’ age and profession, and other concomitant life-threatening injuries. Recent advances in microsurgical techniques have made replantation and revascularization increasingly reliable options for patients who sustain devastating upper-extremity injuries [4-5].

In Pakistan, upper-limb amputations are more common in rural areas and mostly involve younger age groups. The most common causes are chopping machines, followed by grass cutters, heavy machinery, and traffic accidents [6]. There are no published data suggesting the success rates of replantation and revascularization in such cases in Pakistan. However, advances in microsurgery techniques have made replantation a commonly performed surgery in medical centers throughout the world. With increasing knowledge, technology advances, and surgeons’ experience, the success rate has reached 80%-90% [7]. In the past, replantation focused on improving replant survival, but with advances in surgical methods, the aim has shifted to maximizing function and enabling efficient rehabilitation [8].

Replantation cannot be considered to be successful until the function of the amputated segment is restored, which requires various secondary procedures. After limb salvage, a much bigger challenge awaits: functional rehabilitation. These limbs have numerous functional deficits and require more than one secondary procedure to restore near-normal function.

The type of injury (mode and level) is among the most important factors in functional outcomes [9]. Better functional results are achieved with distal and clean-cut injuries than with proximal and crush injuries of the digits [9-10]. Sharply amputated fingers have higher survival rates (91.4%) than crush and avulsion injuries of the fingers (68.4% and 66.3%, respectively) [11]. The outcomes are less predictable in cases of significant tissue damage and longer distances of muscle reinnervation. Amputation at the level of the elbow joint has been reported to result in the most limited range of motion for all upper-extremity amputations due to joint disintegration and soft-tissue injury [12].

Secondary surgeries on replanted and revascularized upper limbs vary based on the extent, mechanism, and level of injury and the adequacy of bone, tendon, nerve, and soft-tissue coverage as well as postoperative rehabilitation [3]. When the patient has had successful reattachment of upper-limb parts but continues to have functional restrictions, the primary surgeon and the patient make the decision whether to perform secondary procedures after detailed evaluation and discussion of these procedures and their outcomes. Recent studies have found that 80%-90% of replantation surgeries result in viable digits; however, these digits often have an imperfect function, and many patients undergoing replantation and revascularization have various secondary procedures to improve function in the amputated body parts [13]. Secondary surgery after replantation and revascularization corrects many common problems, including tendon adhesions, joint stiffness, malunion, contractures, non-healing wounds, and decreased range of motion [14].

Secondary reconstructive surgeries can be grouped into two categories (early and late) according to the type of procedure required by the patient. Commonly performed secondary procedures are tenolysis, neurolysis, nerve transfer, neuroma excision, soft-tissue coverage, joint contracture release, tendon transfer, arthrodesis, and correction of bony nonunion, malunion, and soft-tissue contracture release [10].

Multiple assessment tools are used to assess disability of the upper limb. The Disability of the Arm, Shoulder, and Hand (DASH) questionnaire is an upper-extremity-specific outcome measure developed by the American Academy of Orthopedic Surgeons to assess upper-limb function [15]. It is commonly used in clinical trials and studies on upper-extremity disorders and has been translated into several languages. The Quick DASH (QuickDASH) is a shorter version. The present study used the QuickDASH questionnaire to evaluate the long-term functional outcomes of replanted and revascularized upper limbs after corrective secondary procedures.

## Materials and methods

After approval from the hospital ethical committee, a prospective study was performed in 40 patients (male n=29, female n=11) from January 2015 till December 2018. The sample included all the patients on whom we performed various secondary procedures after successful replantation and revascularization. A total of 62 replantation (n=26) and revascularization (n=38) surgeries was performed over three years. Eight patients with injuries to more than one limb were excluded, bringing the sample to 54. Of these 54 patients, 40 needed corrective secondary procedures to improve function because they failed to achieve more than M3 motor function (moving against partial resistance). The rest of the patients were excluded from the study on the basis of no need for any further secondary procedures following primary replantation and revascularization.

The data collected included the patients’ demographics, mechanism, and level of injury, the number of injured digits and hands, and the number and nature of secondary procedures. The exclusion criteria included incomplete documentation, non-traumatic amputation, lack of follow-up, and more than M3 motor function. All the patients in the study provided informed consent. Details of the various secondary procedures and their functional outcomes were recorded. The patients’ functional outcomes after various secondary procedures were recorded administering the QuickDASH either at the plastic surgery clinic or via the phone. Functional outcomes were assessed by calculating preoperative to postoperative changes in the QuickDASH scores. The follow-up period was kept at six months for final evaluation.

The data analysis was performed using SPSS 22.0 (IBM Corp., Armonk, NY, US). Descriptive statistics were calculated for the qualitative and quantitative variables while measures of central tendency were estimated for the numerical variables. Effect modifiers, such as age, gender, and comorbid conditions, were controlled via stratification. The post-stratification chi-square test and student t-tests were performed for the categorical and numerical variables, respectively. p<0.05 was considered significant.

## Results

A total of 40 patients participated in the study over three years. During this period, 62 replantation and revascularization surgeries were performed on 54 patients, but only 40 patients fulfilled the study’s inclusion criteria. All 40 patients underwent secondary procedures, including about 20 replantation and 25 revascularization surgeries. Significantly more male patients (72.5%, n=29) than female patients (27.5%, n=11, CI 1.13-1.41, p<0.01) underwent secondary procedures. Around 12.5% (n=5) of the 40 patients had injuries to more than two digits or limbs. The patients’ mean age was 30.56 ± 6.89 years. Sample patients in the range of 22.0-40.0 years most commonly (47.5%, n=19) underwent secondary procedures.

**Figure 1:**
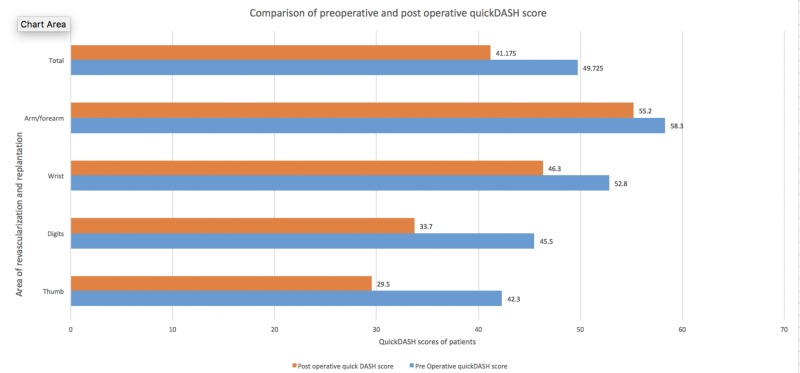
Comparison of preoperative and postoperative QuickDASH score following secondary procedures QuickDASH: Quick disability of the arm, shoulder and hand questionnaire

Among all the patients who underwent secondary procedures, the level of injury was the arm (7.5%, n=3), trans-metacarpal (15%, n=6), elbow and forearm (5%, n=5), thumbs (20%, n=8), digits (35%, n=14), and wrist (17.5%, n=7). The most common injury mechanisms were clean-cut injuries (n=20), followed by crush injuries (n=12) and avulsion injuries (n=8). The mean number of secondary procedures performed per patient was 2.34. Patients with avulsion injuries had an average of 3.17 secondary procedures while those with sharp injuries had an average of 1.38. This difference was statistically significant in the paired samples t-test (CI 1.02-2.37, P=0.0168). The most common secondary procedures in both replantation and revascularization were tenolysis (60%, n=24), followed by soft-tissue coverage, including split- and full-thickness grafting and local and regional flaps (37.5%, n=15); contracture release (32.5%, n=13); tendon transfer (22.5%, n=9); and arthrodesis 25% (n=10). Among injury types, the QuickDASH mean score improvement was the lowest for avulsion injuries, declining from 52.5 preoperation to 49.4 postoperation. The QuickDASH mean score improvement for sharp and crush injuries was 47.5 to 39.6 and 49.2 to 42.5, respectively (Figure 1).

**Figure 2:**
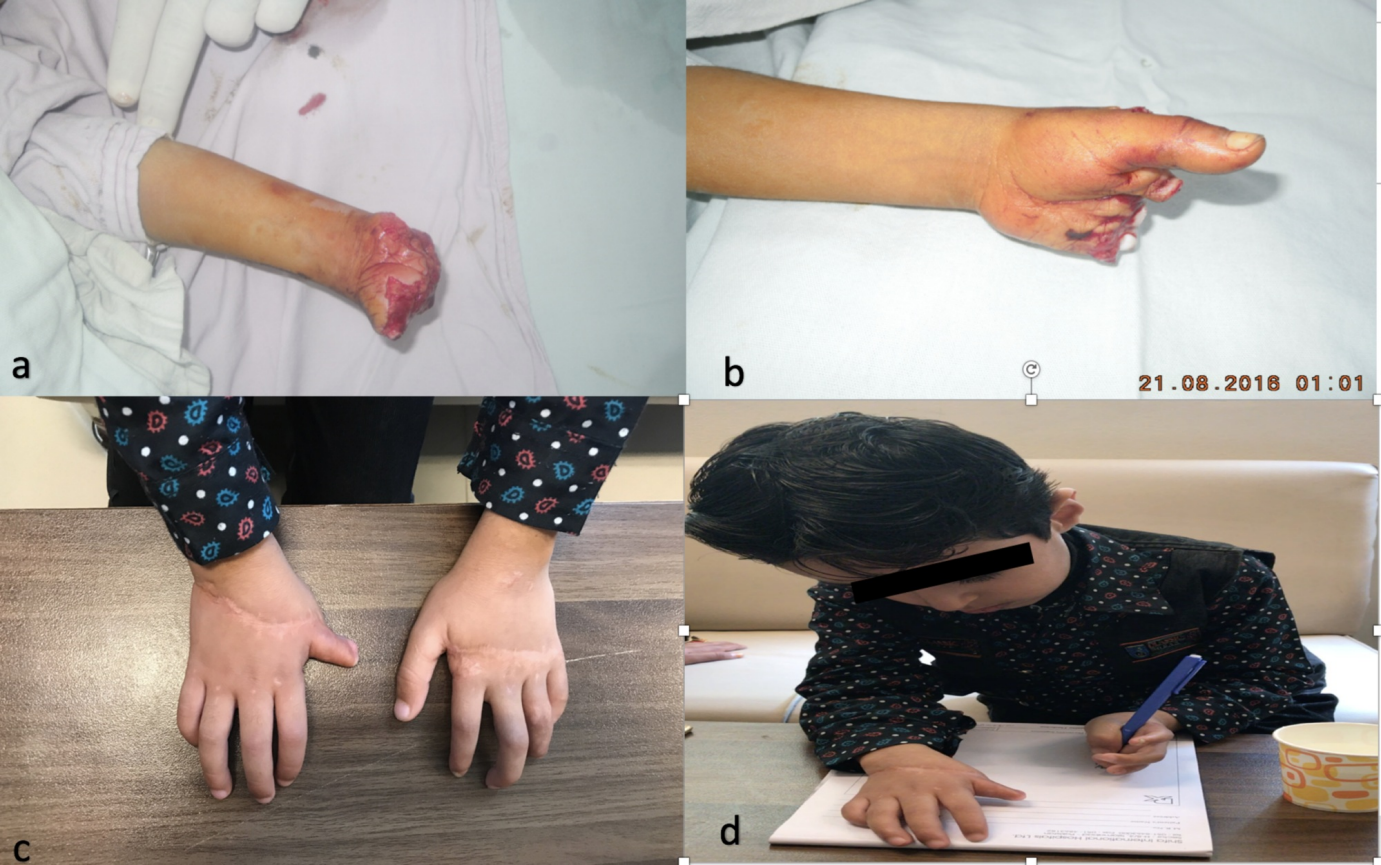
2a and 2b showing bilateral transmetacarpal amputation in a six-year-old boy due to a grass-cutting machine. 2c showing the replanted hands after tenolysis and nerve repair. 2d showing good function achieved after secondary procedures.

The mean QuickDASH score for thumb injuries was 42.3 preoperation and 29.5 after secondary procedures, which was statistically significant (CI 11.12-14.87, p<0.01). Regarding finger injuries, the mean difference was also statistically significant: 45.5 preoperation and 33.7 postoperation (CI 9.89-13.70, p<0.01). For wrist injuries, the QuickDASH mean score was 52.8 preoperation and 46.3 postoperation (CI 1.81-6.58, p=0.0023). The QuickDASH scores of patients with arm and forearm injuries showed no statistically significant improvement, with a decline from 58.3 to 55.2 (p=0.98). The overall replantation and revascularization scores pre and postoperation were 49.725 and 41.175, respectively (CI 8.35-8.75, p<0.01). Overall, the results indicated improvement in the QuickDASH scores after secondary procedures on replantation and revascularization patients. The QuickDASH score improvement was more pronounced for distal injuries than more proximal injuries (Figure 2).

## Discussion

Upper-limb amputations remain functionally and psychological devastating and mostly involve younger and more active age groups. For the majority of patients, these injuries are life-changing events that require multiple surgeries, lengthy hospital stays, financial and psychological constraints, and time away from work [16]. Ever since the first successful upper-limb replantation was reported in 1964, though replantation and revascularization of amputated limbs have allowed surgeons to preserve amputees’ quality of life [17]. Microsurgical instrument advances, improved surgical expertise, well-established microsurgical centers, and safe, lengthy anesthesia have improved survival rates for replantation and revascularization of amputated segments. The survival rate for upper-limb replantation and revascularization in our department is approximately 81%, comparable to the results of various international studies [18-19]. Previously, crush and avulsion injuries were considered to be contraindications for replantation, but improved salvage rates of 90% have become attainable in these devastating injuries [20].

Successful replantation and revascularization is only half of the procedures needed to achieve functional limbs. These limbs have many functional deficits, including adhesions, contractures, poor range of motion, and loss of protective sensation. In addition to good rehabilitation, these patients need one or more secondary procedures to obtain the desired results. The reported incidence of secondary procedures after revascularization and replantation ranges from 15% to 80%, with an average of 50% of cases [21]. Various secondary procedures are needed early (e.g., soft-tissue coverage) and late (e.g., after replantation surgery) in time.

Many tools are available to measure the function of upper extremities, such as the Michigan Hand Questionnaire and Patient-Rated Wrist Evaluation Score [22]. We used QuickDASH to assess our patients’ functional outcomes before and after various secondary procedures because it is reliable and easy to use and can assess any part of the upper limb. A shorter version of the original 30-item DASH questionnaire, the QuickDASH contains 11 only items and is used to interpret patients’ ability to perform certain upper-limb activities [23-25]. On this self-report questionnaire, patients use a five-point Likert scale to rate the level of severity, function, and difficulties with day-to-day life activities [23-24]. The QuickDASH is more convenient to use than the DASH because it places less burden on patients. To calculate Quick DASH scores, at least 10 of the 11 items must be completed; scores cannot be calculated if more than one item is missing [25]. The QuickDASH is as reliable as the original DASH based on a comparison study showing no major differences in their results [25]. Scores are calculated as [(sum of n responses / n) - 1]. x 25, where n is the number of completed responses. Higher DASH scores indicate a greater disability and poorer function.

In our department, secondary procedures were performed as early as three months post-replantation and revascularization when the patients failed to achieve more than M3 motor function on the Medical Research Council grading system. A previous study also similarly included patients with less than M3 motor function [10]. We noted that patients with clean-cut amputations needed fewer secondary procedures than patients with avulsion and crush injuries. Earlier research divided secondary procedures for 11 upper-extremity replantations into early and late procedures. Early procedures included only soft-tissue procedures, such as grafting and skin flaps, and late procedures included tenolysis and tendon transfer [26]. For the sake of convenience, we similarly divided our secondary procedures into early and late procedures.

A review of the functional outcomes of 29 revascularization and replantation surgeries found an average of 4.2 secondary surgical procedures per patients. The analysis included minor secondary procedures such as debridement and flap division [27]. In our study, a mean of 2.34 procedures was required to achieve better functional outcomes. Other studies reported that proximal injuries had poorer functional outcomes. Even though surgery on fingers is more complex, rehabilitation is faster and easier. More proximal injuries have a risk of reperfusion injury due to the much larger muscle bulk and longer distances of reinnervation [28-29]. Our findings accord with these results. According to our study, finger and thumb amputations and revascularization surgeries have better functional outcomes after secondary procedures then proximal-level injuries, with average scores of 29 (thumbs) and 33.5 (fingers). These results correspond to those from earlier research [30].

## Conclusions

This study finds that the level and mechanism of injury are important predictors of the functional outcomes of the replantation and revascularization of amputated upper-limb appendages. Most replanted and revascularized upper limbs have numerous functional limitations, and achieving good functional results requires one or more secondary procedures, whose type depends on various factors such as the injury type and mechanism. The QuickDASH, administered before and after the secondary procedures, is an easy, reliable, and effective way to evaluate patients’ functional outcomes.
